# Prevalence of Intestinal Parasitic Infections and Associated Water, Sanitation, and Hygiene Risk Factors among School Children in Mwea Irrigation Scheme, Kirinyaga County, Kenya

**DOI:** 10.1155/2020/3974156

**Published:** 2020-05-11

**Authors:** Elizabeth Njambi, Dennis Magu, Janet Masaku, Collins Okoyo, Sammy M. Njenga

**Affiliations:** ^1^College of Health Sciences (COHES), Jomo Kenyatta University of Agriculture and Technology (JKUAT), P. O. Box 62000–00200, Nairobi, Kenya; ^2^Eastern and Southern Africa Centre of International Parasite Control (ESACIPAC), Kenya Medical Research Institute (KEMRI), P. O. Box 54840–00200, Nairobi, Kenya

## Abstract

School children bear a significant burden of intestinal parasitic infections. Because they spend most of their time at home and school, it is necessary to identify the key water, sanitation, and hygiene (WASH) factors associated with these infections in both environments. This was a cross-sectional survey conducted in Mwea West, Kirinyaga County. 180 primary school children aged 8–14 years were randomly selected from three schools (Mianya, Mbui Njeru, and Mukou primary schools). Questionnaires and checklists were administered and single stool samples were collected. Stool samples were microscopically examined for *Schistosoma mansoni*, soil-transmitted helminths, and protozoan infections. Data on WASH were obtained at home and school. The factors significantly associated with *S. mansoni* and intestinal protozoa infections in the school children were determined using univariable and multivariable logistic regression models reporting the odds ratio at 95% confidence intervals. The overall prevalence of *S. mansoni* and intestinal protozoa infections was 70.5% (95% CI: 59.1–84.3) and 32.7% (95% CI: 26.8–40.1), respectively. Only one case of STH (*A. lumbricoides*) was identified. The prevalence of coinfections of *S. mansoni* and intestinal protozoa infections was 22.8% (95% CI: 19.2–27.1). An increased prevalence of *S. mansoni* infection was associated with children above 12 years (aOR = 3.19, *p*=0.015), those in Mianya primary (aOR = 1.23, *p*=0.001), those in Mukou primary (aOR = 3.19, *p*=0.001), and reported behavior of wearing shoes at home (aOR = 1.67, *p*=0.010). However, handwashing behavior after defecation at home (aOR = 0.39, *p*=0.001) was protective against *S. mansoni* infection. For any protozoan infection, male children had increased odds of infection (aOR = 2.41, *p*=0.001) while use of wiping material (aOR = 0.55, *p*=0.019) and water contact (aOR = 0.32, *p*=0.001) was protective against intestinal protozoa infections. Infections with *S. mansoni* and any protozoa and their coinfection were present. Findings revealed that several hygiene factors were protective against infections while other were risk factors. Therefore, deworming should be complemented with behavior education on hygienic habits.

## 1. Introduction

Intestinal parasitic infections are a major public health concern in developing countries and the risk factors for infection include living in rural areas, poor communities, poor sanitation, lack of clean water, and poor personal hygiene [1]. In addition to these risk factors, low level of awareness resulting in poor hygiene habits leads to school-aged children suffering the highest infection rates [2, 3]. These infections are caused by soil-transmitted helminths (STH), *Schistosoma mansoni*, and intestinal protozoan parasites among others. STH are commonly caused by infection with *Ascaris lumbricoides* (roundworm), *Ancylostoma duodenale*, *Necator americanus* (hookworm), and *Trichuris trichiura* (whipworm) [4]. Schistosomiasis affects almost 240 million people worldwide, and more than 700 million people live in endemic areas where there is presence of water bodies that harbor susceptible species of snails [5].

Information on the prevalence of intestinal protozoa infections is scarce and little data are available from Sub-Saharan Africa [6]. Studies have shown that *Giardia intestinalis* and *Entamoeba histolytica* are the most common pathogenic intestinal protozoa in temperate and tropical countries, especially among children and the elderly, causing severe diarrhea. Transmission of these pathogenic protozoa is by consumption of contaminated water and food [7, 8]. Globally, an estimated 400 million school children are the worst affected by intestinal parasitic infections and due to the similar nature of transmission cycles, coinfections are common [9, 10]. In Sub-Saharan Africa, an estimated 89.9 million school age children are infected, and studies have shown that factors associated with transmission of infections include fecal contamination of water sources, especially the unimproved sources, lack of environmental sanitation, poor socioeconomic conditions, and poor hygiene practices [9–14].

Transmission of these infections among school children can be divided into two routes: home (domestic domain) and school (public domain) where they spend most of their time [15, 16]. Domestic domain is the geographical location occupied by and under the control of a household while public domain includes public places of work, schooling, commerce, and recreation as well as the streets and fields [16]. Studies have revealed that factors influencing the spread of intestinal parasites were largely centered in the family [16]. In addition, increased risk of transmission has been observed where there was crowding of children (such as schools) due to increased contact and environmental contamination [17].

It has been reported that there is inadequate reliable national data available on the status of school water, sanitation, and hygiene (WASH) in Kenya [18]. In addition, majority of studies performed on WASH and association with infections in school children only captures data from one environment while ignoring other potentially infectious environments [15]. This study determined the prevalence of STH, *S. mansoni,* and intestinal protozoa infections in school children and investigated the WASH factors associated with their occurrence at home and school.

## 2. Materials and Methods

### 2.1. Study Area and Population

The study was conducted in Mwea West Sub-County, Kirinyaga County, in Central Kenya. The Mwea irrigation scheme is endemic for *S. mansoni* and STH infections, thus making it an ideal study site [19, 20]. The county is located about 150 km northeast of Nairobi. It covers an area of 513 km^2^; it is estimated to have 51,444 households and a total population of 176,261 people. There are 58,970 school-aged children in Mwea [21]. The main socioeconomic activity in this area is rice farming, which is done through canal irrigation using water from rivers Thiba and Nyamindi. In this study, school-going children aged 8 to 14 years from three primary schools in Mwea West Sub-County were included.

### 2.2. Study Design, Sample Size, and Sampling Strategy

This was a cross-sectional study and a total of 60 children per school were randomly sampled. Sampling was based on the World Health Organization (WHO) guidelines for STH and schistosomiasis surveys and preventive chemotherapy for STH, specifically school-based deworming (SBD) programs [4, 22, 23]. The guideline proposed that, for surveys, a few schools near irrigated areas should be selected and in each school fifty children from the three upper classes should be selected and asked to provide single stool sample. In line with this guideline, three schools in the study area (Mianya, Mukou, and Mbui Njeru primary schools) were purposively selected based on previous studies showing prevalence of *S*. *mansoni* and STH [20, 24]. In each school, we randomly sampled 60 children aged 8 to 14 years, who were presumably in classes 2 to 6. This study included children of this age group because recent studies have showed that intestinal parasites occur mainly in children among this age group [24]. All the selected children provided single stool samples and participated in both the school and household components of the study.

### 2.3. Field Procedures

The school component of the study was carried out between morning and midmorning hours and the household component was carried out between late afternoon and evening on the same day (15:30 to 18:00 hours). Data collection using piloted questionnaires and checklists was carried out with the help of two trained field assistants familiar with the study area. The field assistants were also responsible for following up the school children to their homes after school.

### 2.4. School Survey

The research team visited each school prior to the survey and participants were selected using generated random numbers. Parents or guardians of the selected children were invited to a meeting to communicate the study purpose and obtain their consent. All parents/guardians consented to allow their children to participate in the study and a written informed consent was obtained from them before conducting the study. In addition, assent was sought (verbal) from participating children who were above 13 years. Each participating child was left with a small plastic container (polypot) and instructed to collect a morning stool sample on the day of the survey, and the plastic containers containing morning stool samples were collected from each child. Upon receipt of the stool sample, each of the stool container was labelled with the participant's unique identification number. The samples were kept in a cooler box and transported to Kimbimbi Sub-County hospital laboratory for microscopic screening within the same day. Thereafter, each child responded to an interviewer-administered questionnaire on WASH which had been pretested at a local school in Mwea and was adapted before administration.

### 2.5. Household Survey

Using a checklist, direct observations were carried out at each participating child's home to investigate the water sources and sanitation facilities. This was performed with the assistance of the head of household or any adult family member (above 18 years old).

### 2.6. Data Collection Tools

A questionnaire was developed to obtain information on children's demographic data, hygiene habits, eating and playing habits, handwashing behavior at critical times, and use of water and sanitation facilities. Direct observations on water sources and sanitation facilities were carried out at the school compound and at the homes of the children. Water sources and sanitation facilities were defined using the Joint Monitoring Programme (JMP) guidelines of the WHO and the United Nations Children Fund (UNICEF) for water and sanitation [25]. Improved sanitation facilities were defined as availability of flush toilet, toilet connected to a piped sewer system, toilet connected to a septic system, flush to a pit latrine, ventilated improved pit latrine (VIP), and pit latrine with slab and composting toilet. Improved water sources were defined as availability of piped water into dwelling, piped water into yard/plot, public tap or stand pipe, borehole, protected well or spring, bottled water, and rain water. The condition of the toilets (cleanliness) was also observed as well as presence of handwashing facilities and anal cleansing material in the toilets.

### 2.7. Laboratory Procedures

The specimens were checked for identification number, quantity, and quality (no urine or dirt) and divided into two aliquots. One aliquot was used for microscopic screening of *S. mansoni* and STH infections using double slide Kato-Katz technique while the other was used to screen for protozoan infection using direct saline and iodine wet mount methods. Quality of reagents and instruments was checked by laboratory technicians.

### 2.8. Helminths Screening

Each stool specimen was analyzed for *S. mansoni* and STH infections using the double slide Kato-Katz thick smears method [26]. Briefly, the stool sample was passed through a metal sieve to remove fibrous material. Using a spatula, some amount of stool was collected and filled in a template on a slide. Cellophane soaked in glycerine malachite green was placed on the smear and the slide was turned upside down, pressed, and allowed to spread evenly. After a clearance time of 30 minutes, the slide was examined under a light microscope (100x magnification). Egg counts per slide was multiplied by a factor of 24 to obtain eggs per gram of feces (epg) [22].

### 2.9. Protozoa Screening

For each sample, two wet preparations were made for saline wet mount and iodine wet mount tests [27, 28]. For each of the preparations, approximately 2 grams of stool sample was picked up using a wooden stick and placed on two separate glass slides. The first preparation was mixed with a drop of normal saline (0.9%) and the second with a drop of dilute Lugol's iodine and normal saline (1 : 5 distilled water). Both slides were covered with a cover slip and observed under the microscope at 10x and at 40x magnifications. Saline preparation and iodine allowed for visualization of motile trophozoites and cysts, respectively.

### 2.10. Data Management and Statistical Analysis

Data collected were checked to ensure accuracy and completeness on site before double entry into a Microsoft Excel spreadsheet. The observed prevalence of *S. mansoni* and intestinal protozoa infections were calculated by school, as well as by demographic variables, and the 95% confidence intervals (CIs) were obtained using binomial logistic regression, taking into account clustering by schools. For purposes of this analysis, the following age groups were used: <10, 10–12, and >12 years. Using univariable analysis, factors associated with *S. mansoni* and intestinal protozoa infections were determined and described as odds ratio (OR). Minimum adequate variables for multivariable analysis were selected by specifying an inclusion criterion of *p* value <0.05 in a sequential (block-wise) variable selection method. Adjusted odds ratios (aOR) were obtained by mutually adjusting all minimum generated variables using a multivariable logistic regression model. All statistical analyses were carried out using STATA version 14.1 (STATA Corporation, College Station, TX, USA).

## 3. Results

### 3.1. Sociodemographic, Household, and School Characteristics

The overall data were collected from 180 children in three primary schools in Mwea West. The mean age of children was 10.0 years (range: 7–15 years, standard deviation (SD) = 1.6 years). There was an equal representation of study participants in terms of gender (50.0%/50.0%) females/males. Majority of the study participants were in the age group 10–12 years (99 participants; 55%), followed by those below 10 years (66 participants; 36.6%), and those above 12 years (15 participants; 8.3%).

Out of all the households surveyed, 35.6% (64 households) used improved water sources and 152 (84%) used improved latrines (VIP). All three schools used improved sources of water for drinking and had improved latrine facilities (VIP). For personal and environmental hygiene purposes such as handwashing and cleaning classrooms, Mianya and Mbui Njeru used water from unimproved sources (surface water) while Mukou used piped water (improved sources). The pupil per latrine ratio was 23 : 1, 67 : 1, and 17 : 1 for Mianya, Mbui Njeru, and Mukou primary schools, respectively.

### 3.2. Prevalence of *S. mansoni* and STH Infections

**Figure 1:**
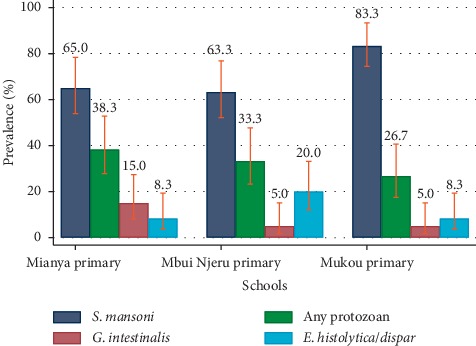
Prevalence of intestinal parasitic infections in school children per school.

Only one case of STH (*A. lumbricoides*) was identified. The overall prevalence of *S. mansoni* was 70.5% (95% CI: 59.0%–84.2%). The mean infection intensity was 376.2 epg (95% CI: 222.3–530.1) and was categorized in overall as moderate infection. Majority of the infections were light infections 46 (25.6%), followed by moderate infections 41 (22.8%) and severe infections 40 (22.2%). Out of the three schools, Mukou primary had the highest prevalence of infection, 83.3% (95% CI: 74.4.4–93.3, *p*=0.002), followed by Mianya primary, 65.0% (95% CI: 53.9–78.3, *p*=0.001) and Mbui Njeru primary, 63.3% (95% CI: 52.2–76.8, *p*=0.001) (Figure 1). Children aged above 12 years had a higher prevalence of 86.6% (95% CI: 81.4–98.3, *p*=0.001), followed by those aged 10 to 12 years, 71.7% (95% CI: 54.5–94.4, *p*=0.018), and those below 10 years, 65.2% (95% CI: 49.4–85.9, *p*=0.002). The male gender reported a higher prevalence of infection compared to the female gender, 73.3% (95% CI: 62.9–82.1, *p*=0.001) and 67.7% (95% CI: 57.0–77.2, *p*=0.002), respectively (Table 1).

### 3.3. Prevalence of Intestinal Protozoa Infection

The overall prevalence of protozoan infections was 32.7% (95% CI: 26.7–40.1). Out of the three schools, Mianya primary had a higher prevalence of infection, 38.3% (95% CI: 27.8–52.8, *p*=0.001), followed by Mbui Njeru, 33.3% (95% CI: 23.3–47.7, *p*=0.001), and Mukou primary, 26.7% (95% CI: 17.5–40.6, *p*=0.0001) (Figure 1). A slightly higher prevalence was reported in females compared to males, 33.3% (95% CI: 23.7–44.0, *p*=0.001) and 32.2% (95% CI: 22.7–42.9, *p*=0.001), respectively. Children aged between 10 to 12 years had the highest prevalence, 34.3% (95% CI: 22.3–52.9, *p*=0.001), followed by those above 12 years, 33.3% (95% CI: 21.2–52.4, *p*=0.003), and those below 10 years, 30.3% (95% CI: 19.3–47.6, *p*=0.001). The most prevalent species was *Entamoeba coli* infections, 18.8% (95% CI: 17.8–20.0, *p*=0.001), followed by *E*. *histolytica/dispar* infections, 12.2% (95% CI: 6.5–22.8, *p*=0.001), and *G. intestinalis* infection, 8.3% (95% CI: 3.8–18.2, *p*=0.001) (Table 1).

### 3.4. Prevalence of Coinfections

The overall prevalence of *S. mansoni* and any protozoa infection was 22.8% (95% CI: 19.2–27.1). Prevalence in Mianya primary was the highest, 26.7% (95% CI: 17.5–40.6, *p*=0.001), then Mukou primary, 21.7% (95% CI: 13.4–35.1, *p*=0.001), and Mbui Njeru primary, 20.0% (95% CI: 12.1–33.2, *p*=0.001). Coinfections were highest among those aged above 12 years, 33.3% (95% CI: 21.2–52.4, *p*=0.003), followed by those aged below 10 years, 24.2% (95% CI: 14.0–41.9,*p*=0.001), and those between 10–12 years, 20.2% (95% CI: 16.1–25.3, *p*=0.001). Also, female children had a greater prevalence compared to males, 25.6% (95% CI: 16.3–40.1, *p*=0.001) and 20.0% (95% CI: 16.6–24.2, *p*=0.001), respectively.

### 3.5. Univariable Analysis of Risk Factors

Factors associated with greater odds of *S. mansoni* infection were pupils at Mukou primary school (OR = 2.69, *p*=0.024), children aged above 12 years (OR = 3.48, *p*=0.002), and handwashing after defecation at school (OR = 1.22, *p*=0.001). Handwashing after defecation at home (OR = 0.48, *p*=0.001) was protective for *S. mansoni* infection. Pupils at Mbui Njeru and Mukou primary (OR = 0.80, *p*=0.001) (OR = 0.58, *p*=0.001), respectively had lower odds of protozoa infections. Also, use of wiping material (OR = 0.64, *p*=0.001), contact with water bodies (OR = 0.70, *p*=0.001), and presence of a toilet handwashing set-up (OR = 0.78, *p*=0.034) while adequately cleaned toilets at home slightly increased the risk of protozoan infections (OR = 1.84, *p*=0.040) (Table 2).

### 3.6. Multivariable Analysis of Risk Factors

Factors-associated increased prevalence of *S. mansoni* infection was children above 12 years (aOR = 3.19, *p*=0.015), Mianya primary (aOR = 1.23, *p*=0.001), Mukou primary (aOR = 3.19, *p*=0.001), and reported behavior of wearing shoes at home (aOR = 1.67, *p*=0.010). Handwashing behavior after defecation at home (aOR = 0.39, *p*=0.001) was protective against *S. mansoni* infection. For any protozoan infection, male children had increased odds of infection (aOR = 2.41, *p*=0.001) while use of wiping material (aOR = 0.55, *p*=0.019) and water contact (aOR = 0.32, *p*=0.001) was protective against intestinal protozoa infections (Table 3).

Variables selected for *S. mansoni* infection: school, age group, gender, handwashing after defecation at home, type of wiping material used, and handwashing after defecation at school.

Variables selected for any protozoan infection: school, gender, use of wiping material, contact with water bodies, presence of a toilet handwashing station, and clean latrines.

## 4. Discussion

In Kenya, schistosomiasis caused by *S. mansoni* occurs mostly in the central and western parts of the country [29, 30], and in cases where *S. mansoni* infections are a public health concern, the WHO recommends for mass drug administration (MDA) [22]. In this study, overall prevalence of *S. mansoni* was higher compared to findings from other past studies conducted in the study area. In 2015, prevalence of *S. mansoni* infection was 53.7% while in 2012 it was 47.4% [24, 31], respectively. This shows that reinfection is on the rise after withdrawal of MDA. Therefore, the study population requires consistent annual treatment campaigns [22]. In the Mwea irrigation scheme, high prevalence of *S. mansoni* can be attributed to rich farming which leads to repeated contact with water bodies where snails, the intermediate hosts of schistosomiasis, breed [32]. Further, due to lack of sanitation facilities in the farms, the water in irrigation canals is contaminated with feces and therefore contact with the water leads to transfer of infections [33].

A significantly higher prevalence of *S. mansoni* was observed in children above 12 years and these findings can be explained by the fact that children in this age group are more involved in household chores, farming, or recreational activities potentially bringing them in contact with water [34]. Similar findings were observed in a study in Western Kenya where prevalence peaked in early adolescence (*p* < 0.003) [35]. This present study did not reveal gender as a risk factor for *S. mansoni* infection. However, male school children had slightly greater odds of infection as compared to female children. This may be due to the playful habits of boys compared to girls which may put them in contact with infected water. This corroborates findings of a study conducted in the same area which reported a higher prevalence in males than females [24].

As has been mentioned earlier, information on the prevalence of intestinal protozoa infections is scarce and little data are available from Sub-Saharan Africa [6]. Therefore, determining the prevalence of these infections in school children was a key objective. The observed prevalence of intestinal protozoa was similar to findings in a study conducted in Thika town, Central Kenya, 32.7% and 38.9%, respectively [36]. Similarly, in the Thika study, the presence of infections from pathogenic species was also identified, specifically *Entamoeba histolytica*/*dispar* (14.6%) and *Giardia lamblia* (6.9%). Findings from a study in Murang'a County in Central Kenya also observed infections with intestinal protozoa among school-aged children [37]. This shows that infections with intestinal protozoa infections are overlooked and should be considered in future survey and MDA activities.

Coinfections with *S. mansoni* and any intestinal protozoa infection were also observed among the primary school children. Multiple infections are common probably because of similarities in modes of transmission. Among primary school children in Murang'a County, prevalence of coinfection was 9.3% and the most frequent combination was between *E. histolytica* and *A. lumbricoides* [37]. Similarly, multiple infections in Ethiopian school children were 6.2% and *S. mansoni* and intestinal protozoa coinfection was observed although to a smaller degree [38]. Infections with intestinal helminths are a common occurrence among primary school children in Kenya with ascariasis, trichuriasis, and hookworm infections as the most common [31, 36, 39]. However, in this study, only one case of ascariasis was detected. This may be because the Kato Katz technique used for screening of STH infection was not a sensitive method and may have missed light infections.

In order to reduce reinfection, there is need to focus on WASH factors which have been associated with intestinal parasitic infections. These infections may occur at home or at school because children spend most of their time in these environments [15, 16]. This study investigated WASH factors associated with infections at homes and schools. In the study, the behavior of handwashing after defecation at home was protective against *S. mansoni* infections. This agrees with studies that have shown hygienic habits, such as handwashing, which play a significant role in preventing transmission of these infections [40]. However, wearing shoes in school children at home was associated with increased risk of *S. mansoni* infections. This may be because children did not consistently wear shoes, therefore increasing exposure to infected snails.

The study found that male children had increased odds of intestinal protozoa infections than the females. A study in Ethiopian primary school children observed the same association [38]. This higher prevalence may be attributed to the adventurous nature of male children which increases their risk of infections when they are outdoors as compared to females. Also, the use of wiping material was found to be protective against intestinal protozoa and this reaffirms that hygienic behaviors are significant in preventing transmission. Water contact was also seen as protective against intestinal protozoa. The transmission of intestinal protozoa infections is via fecal-oral route and therefore contact water would not be a risk for these infections [41]. Studies have shown that open defecation has been significantly associated with intestinal parasitic infections (including *E. histolytica/dispar*) [42, 43]. A study in West Africa revealed that *E. histolytica/dispar* had a prevalence of 14.4% and open defecation was common [43]. Similar findings were observed in the current study although there was no significant association.

Pupil-to-latrine ratio is used to determine whether a school's sanitation facilities are adequate. This has been associated with increased toilet use which leads to better sanitation conditions interrupting transmission of infections [44]. According to the Kenyan government, the recommended pupil-to-latrine ratio is 25 : 1 and 30 : 1 for girls and boys, respectively [45]. Out of the three schools, Mianya and Mukou primary schools met the required pupil-to-latrine ratio for both girls and boys. Mbui Njeru primary did not meet the recommendation and school children may have an increased risk of infection. Therefore, construction of additional latrines is necessary in order to meet the government's recommended ratio [46].

## 5. Limitations

Information on hygiene behavior in school children was self-reported which may be subjective and unreliable compared to observing these behaviors. The WASH factors at school were only collected from three schools and may therefore be inadequate to make inferences. The laboratory methods used for the detection of the intestinal parasitic infections were of limited sensitivity and may have underestimated the prevalence of infections. Identification of the pathogenic *E. histolytica* from the enteric commensals *E. dispar* was not differentiated due to lack of laboratory facilities in the department.

## 6. Conclusion

Infections with *S. mansoni* and intestinal protozoa as well as their coinfection are a public health problem in school children in the Mwea irrigation scheme. The hygiene behavior of handwashing at home was protective against *S. mansoni* infections while use of wiping material and water contact was protective against any protozoan infection. However, wearing shoes at home was a risk factor for *S. mansoni* infection. These findings show that WASH factors at the household level have a significant role in transmission. Also, consistency of deworming for *S. mansoni* infections and treatment for intestinal protozoa infections are necessary. These treatments should be complemented with improvements in school and household WASH conditions and behavior education on hygienic habits.

## Figures and Tables

**Table 1:** Prevalence of *S. mansoni* and intestinal protozoa infections by categories, age, sex, and school.

	*S. mansoni* *n*/*N* (%)	Any intestinal protozoan *n*/*N* (%)	*G. intestinalis* *n*/*N* (%)	*E. histolytica/dispar* *n*/*N* (%)	*E. coli* *n*/*N* (%)
Overall prevalence	127 (70.5%)	59 (32.7%)	15 (8.3%)	22 (12.2%)	34 (18.9%)

Prevalence by age					
<10 years	65.10%	30.30%	5 (7.6%)	7 (10.6%)	12 (18.2%)
10–12 years	71.70%	34.30%	10 (10.1%)	14 (14.1%)	19 (19.2%)
>12 years	86.60%	33.30%	0	1 (6.7%)	3 (20.0%)

Prevalence by gender					
Male	73.30%	32.20%	7 (7.8%)	9 (10.0%)	16 (17.8%)
Female	67.70%	33.30%	8 (8.9%)	13 (14.4%)	18 (20.0%)

Prevalence by school					
Mianya	65.00%	38.30%	9 (15.0%)	5 (8.3%)	11 (18.3%)
Mbui Njeru	63.30%	33.30%	3 (5.0%)	12 (20.0%)	11 (18.3%)
Mukou	83.30%	26.60%	3 (5.0%)	5 (8.3%)	12 (20.0%)

**Table 2:** Univariable analysis of risk factors of *S. mansoni* and intestinal protozoa infections.

Characteristic	*N* = 180, *n* (%)	Univariable analysis [OR, *p*-value]				
		*S. mansoni* (*n* = 127)	Any protozoan (*n* = 59)	*G. intestinalis* (*n* = 15)	*E. histolytica/dispar* (*n* = 22)	*E. coli* (*n* = 34)
Demographics						
School						
Mianya	60 (33.3)	Reference				
Mbui Njeru	60 (33.3)	0.94, *p*=0.849	0.80, *p*=0.001^*∗*^	0.29, *p*=0.081	2.75, *p*=0.075	1.00, *p*=1.000
Mukou	60 (33.3)	2.69, *p* = 0.024^*∗*^	0.58, *p*=0.001^*∗*^	0.29, *p*=0.081	1.00, *p*=1.000	1.11, *p*=0.817
Age group						
<10 years	66 (36.7)	Reference				
10–12 years	99 (55.0)	1.36, *p*=0.634	1.20, *p*=0.746	1.37, *p*=0.737	1.39, *p*=0.591	1.07, *p*=0.949
>12 years	15 (8.3)	3.48, *p*=0.002^*∗*^	1.15, *p*=0.832	—	0.60, *p*=0.604	1.13, *p*=0.456
Gender						
Female	90 (50.0)	Reference				
Male	90 (50.0)	1.31, *p*=0.429	0.95, *p*=0.817	0.86, *p*=0.884	0.66, *p*=0.001^*∗*^	0.86, *p*=0.056
Individual and household characteristics						
Open defecation behavior	90 (50.8)	1.17, *p*=0.410	0.66, *p*=0.227	0.62, *p*=0.001^*∗*^	0.34, *p*=0.039^*∗*^	0.60, *p*=0.036^*∗*^
Use of wiping material	63 (70.8)	1.56, *p*=0.550	0.64, *p*=0.001^*∗*^	2.16, *p*=0.389	0.18, *p*=0.001^*∗*^	0.61, *p*=0.011^*∗*^
Contact with water bodies	160 (88.9)	0.78, *p*=0.659	0.70, *p*=0.001^*∗*^	1.82, *p*=0.493	0.76, *p*=0.212	0.49, *p*=0.161
Eating unwashed food/fruits	46 (25.6)	1.25, *p*=0.784	1.12, *p*=0.673	0.71, *p*=0.020^*∗*^	1.11, *p*=0.847	0.87, *p*=0.796
Handwashing after defecation at home	163 (90.6)	0.48, *p*=0.001^*∗*^	0.51, *p*=0.349	0.37, *p*=0.141	0.40, *p*=0.144	1.09, *p*=0.950
Handwashing before eating at home	161 (89.4)	1.12,*p*=0.234	0.82, *p*=0.178	0.43, *p*=0.250	0.71, *p*=0.628	1.27, *p*=0.629
Behavior of wearing shoes at home	162 (90.5)	1.77, *p*=0.036	0.89, *p*=0.745	0.65, *p*=0.063	2.38, *p*=0.058	0.74, *p*=0.543
Presence of wiping material in the latrine	57 (31.7)	0.86, *p*=0.543	0.92, *p*=0.808	1.49, *p*=0.179	0.59, *p*=0.198	1.04, *p*=0.932
Type of wiping material						
Toilet paper	26 (45.6)	Reference				
Leaves	2 (3.5)	0.37, *p*=0.002^*∗*^	—	—	—	—
Newspaper	29 (50.9)	0.70, *p*=0.050	0.85, *p*=0.708	1.92, *p*=0.270	0.57, *p*=0.001^*∗*^	0.43, *p*=0.132
Presence of a toilet handwashing station	163 (90.6)	1.42, *p*=0.256	0.78, *p*=0.034^*∗*^	0.41, *p*=0.161	1.62, *p*=0.095	0.85, *p*=0.376
Damaged toilet structure	32 (17.8)	1.61,*p*=0.418	0.63, *p*=0.412	1.17, *p*=0.846	0.70, *p*=0.497	0.56, *p*=0.248
Clean latrines	137 (76.1%)	1.21,*p*=0.672	1.84, *p*=0.040^*∗*^	—	1.47, *p*=0.388	1.26, *p*=0.155
Improved sources of water	65 (36.1)	1.45, *p*=0.265	1.08, *p*=0.584	1.19, *p*=0.619*p*	1.56, *p*=0.203	1.12, *p*=0.537
Taken deworming medication	133 (73.9)	1.74, *p*=0.094	0.81, p=0.745	1.45, *p*=0.692	0.46, *p*=0.056	0.68, *p*=0.243
Handwashing after defecation at school	155 (86.6)	1.22, *p*=0.001^*∗*^	0.98, *p*=0.988	1.00, *p*=0.984	0.47, *p*=0.571	1.20, *p*=0.907
Handwashing before eating at school	159 (88.3)	0.95, *p*=0.870	0.97, *p*=0.960	0.85, *p*=0.433	0.81, *p*=0.722	2.39, *p*=0.574
Behavior of wearing shoes at school	158 (88.3)	0.94, *p*=0.876	0.32, *p*=0.113	0.85, *p*=0.843	0.82, *p*=0.625	0.32, *p*=0.039^*∗*^

^*∗*^Significant association (*p* < 0.05); —variable omitted because of insufficient observations.

**Table 3:** Multivariable analysis of risk factors of *S. mansoni* and any intestinal protozoa infection.

Characteristic	Multivariable analysis (aOR, *p* value)	
	*S. mansoni* (*n* = 127)	Any protozoan (*n* = 59)
Demographics		
School		
Mianya	1.23, *p*=0.001^*∗*^	2.03, *p*=0.001^*∗*^
Mbui Njeru	Reference	Reference
Mukou	3.19, *p*=0.001^*∗*^	0.55, *p*=0.001^*∗*^

Age group		
<10 years	Reference	
10–12 years	1.37, *p*=0.649	—
>12 years	3.19, *p*=0.015^*∗*^	—

Gender		
Female	Reference	
Male	1.29, *p*=0.455	2.41, *p*=0.001^*∗*^

Individual and household characteristics		
Use of anal wiping material	—	0.55, *p*=0.019^*∗*^
Handwashing after defecation at home	0.39, *p*=0.001^*∗*^	—
Water contact	—	0.32, *p*=0.001^*∗*^
Behavior of wearing shoes at home	1.67, *p*=0.010^*∗*^	—

^*∗*^Significant association (*p* < 0.05).

## Data Availability

The datasets used to support the findings of this study are available from the corresponding author upon request.

## Abbreviations

aOR: Adjusted odds ratio
BCC: Behavior change communication
CI: Confidence interval
epg: Eggs per gram of feces
ESACIPAC: Eastern and Southern Africa Centre of International Parasite Control
KEMRI: Kenya Medical Research Institute
MDA: Mass drug administration
OR: Odds ratio
SBD: School-based deworming
SD: Standard deviation
SERU: Scientific Ethics and Review Unit
STH: Soil-transmitted helminths
WASH: Water, sanitation, and hygiene
WHO: World Health Organization.
